# Maternal vitamin D levels during pregnancy and offspring schizophrenia

**DOI:** 10.1016/j.schres.2024.06.039

**Published:** 2024-06-29

**Authors:** Andre Sourander, Subina Upadhyaya, Heljä-Marja Surcel, Susanna Hinkka-Yli-Salomäki, Keely Cheslack-Postava, Sanju Silwal, Ian W. McKeague, Alan S. Brown

**Affiliations:** a University of Turku, Department of Child Psychiatry, Research Centre for Child Psychiatry, Turku, Finland; b Turku University Hospital, Department of Child Psychiatry, Turku, Finland; c University of Turku, INVEST Research Flagship, Finland; d Faculty of Medicine, University of Oulu, Finland; e Biobank Borealis of Northern Finland, Oulu, Finland; f Department of Psychiatry, New York State Psychiatric Institute, Columbia University Irving Medical Center, New York, NY, USA; g Department of Biostatistics, Columbia University Mailman School of Public Health, New York, NY, USA; h Department of Epidemiology, Columbia University Mailman School of Public Health, New York, NY, USA

**Keywords:** Maternal, Pregnancy, Antenatal, Vitamin D, Schizophrenia, Offspring

## Abstract

**Background:**

Findings from previous studies on maternal 25(OH)D levels during pregnancy and offspring schizophrenia are limited and inconsistent.

**Methods:**

We used nationwide population-based register data with a nested case-control design to examine the association between maternal 25(OH)D levels during pregnancy and offspring schizophrenia. The cases of schizophrenia (*n* = 1145) were born from 1987 to 1997, and received a diagnosis of schizophrenia by 2017, and were matched with equal number of controls. A quantitative immunoassay was used to measure maternal 25 (OH)D in archived maternal serum in the national biobank of the Finnish Maternity Cohort, collected during the first and early second trimesters. Conditional logistic regression models examined the association between maternal 25(OH)D levels and offspring schizophrenia.

**Results:**

No significant association was found between log-transformed maternal 25(OH)D levels and schizophrenia in unadjusted (OR 0.96, 95 % CI 0.78–1.17, *p* = 0.69) or adjusted analyses (aOR 0.98, 95 % CI 0.79–1.22, *p* = 0.89). Analyses by quintiles also revealed no association between the lowest quintile of maternal 25(OH)D levels and schizophrenia (OR 1.09, 95 % CI 0.81–1.45, *p* = 0.55; aOR 1.06, 95 % CI 0.78–1.45, *p* = 0.71). Maternal 25(OH)D levels, measured in categories, either in deficient category (OR 1.07 (0.85–1.35), *p* = 0.52; aOR 1.05 (0.81–1.34), *p* = 0.88) or insufficient category (OR 1.13, 95 % CI 0.92–1.40, *p* = 0.23; aOR 1.13, 95 % CI 0.90–1.41, *p* = 0.27) were also not associated with offspring schizophrenia.

**Conclusions:**

Maternal vitamin D levels in early pregnancy were not associated with offspring schizophrenia. Future studies measuring vitamin D during different stages of gestation are needed to draw firm conclusions.

## 1. Introduction

Schizophrenia is a severe complex mental disorder characterized by hallucinations, delusions and cognitive deficits, with a lifetime risk of about 1 % and heritability estimated at up to 80 % (Hilker et al., 2018; Sullivan et al., 2003). The etiology of schizophrenia is multifactorial and both genetic and environmental risk factors influence in the development of this disorder (Misiak et al., 2018). The increased risk of schizophrenia has been associated with season of birth (Davies et al., 2003), migrant status (Henssler et al., 2020), and variation in geographical latitudes (Saha et al., 2006), and all these factors are also associated with vitamin D deficiency (Eggemoen et al., 2016; Hauta-Alus et al., 2018).

Vitamin D is crucial for fetal brain development, and maternal nutritional deficiency is linked with several neuropsychiatric disorders including schizophrenia (Cortés-Albornoz et al., 2021; Mattei and Pietrobelli, 2019). Vitamin D receptors expressed abundantly in brain regions in the hypothalamus and dopaminergic neurons, provides more evidence on the importance of vitamin D on central nervous system and mental health (Eyles et al., 2005). Only four previous studies examining the association between maternal vitamin D levels during pregnancy or in newborns using sera samples and offspring schizophrenia, schizoaffective disorder or psychotic symptoms have yielded inconclusive findings. Two studies did not show any association between low maternal vitamin D levels during pregnancy and schizophrenia or schizoaffective disorder (McGrath et al., 2003) and psychotic symptoms (Sullivan et al., 2013), and were based on small sample sizes. Two register-based studies from Denmark with larger sample sizes examined vitamin D in neonatal dried blood samples (Eyles et al., 2018; McGrath et al., 2010), and found positive associations. The first Danish study with 424 case control pairs found a non-linear relationship between vitamin D levels and schizophrenia (increased risk for schizophrenia in the three lowest quintiles and the highest quintile, as compared to fourth quintile) (McGrath et al., 2010). However, another Danish study with 1301 case control pairs found increased risk for schizophrenia for the lowest quintile only when compared to the highest quintile (Eyles et al., 2018).

Two of the previous studies that reported negative results were based on small sample sizes and offered limited adjustment for confounders (McGrath et al., 2003; Sullivan et al., 2013). The two Danish studies showing positive associations examined neonatal vitamin D levels, but did not examine vitamin D levels in early pregnancy (Eyles et al., 2018; McGrath et al., 2010). The present study is the first to examine maternal vitamin D deficiency, measured as circulating 25(OH)D levels in early pregnancy and diagnosed offspring schizophrenia in a large nationwide population-based sample.

The Finnish Prenatal Study of schizophrenia (FiPS-S), is based on a nested case-control study design. The study offers several strengths, including assessment of maternal 25 (OH)D levels based on prospectively collected maternal sera in first and second trimesters, a large sample of schizophrenia cases, and information from several nationwide registers facilitating adjustment for wide range of potential confounding factors. Moreover, the information on maternal vitamin D levels were collected from one of the northernmost European countries in the world, Finland, with little sun exposure during the winter, and before the national recommendation for vitamin D supplementation during pregnancy started in 2004. The consequent vulnerability to vitamin D deficiency during the study period allowed for increased exposure, further increasing power to investigate the association. The aim of this population-based study was to examine maternal 25(OH)D levels in early pregnancy and diagnosed schizophrenia cases among the offspring.

## 2. Methods

The FiPS-S is a nested case control study including all singleton live births in Finland from 1987 to 1997, and followed up for schizophrenia diagnoses in the Care Register for Health Care (CRHC) until 2017.

### 2.1. Finnish maternity cohort

Finnish Maternity Cohort (FMC) consists of approximately 2 million maternal serum samples collected during the first and early second trimesters of pregnancy (5th to 95th percentile: months 2–4 of pregnancy) from over 950,000 gravidae. The FMC comprises archived prenatal serum specimens drawn for routine screening for congenital infections, virtually including all pregnancies in Finland. A protected biorepository at Biobank Borealis in Oulu, Finland stores the serum samples (approximately 1–3 mls of serum from each pregnancy) at −25 °C, and are available for scientific research, after obtaining the informed consent from the pregnant mothers. Finnish nationwide registers were linked with all FMC samples by the unique personal identification code, assigned to all residents in Finland since 1971.

### 2.2. Nationwide registers

The data in the study are derived from three Finnish nationwide registers. The CRHC includes all public and private inpatient diagnoses since 1967 and outpatient diagnoses from specialized services since 1998. The diagnoses are based on the International Classification of Diseases (ICD): ICD-8 from 1969 to 1986, ICD-9 from 1987 to 1995, and ICD-10 since 1996. The Finnish Maternity Birth Register (FMBR), established in 1987 contains comprehensive data on numerous variables relating to pregnancy and the postpartum/neonatal periods (up to 7 days of age) on all pregnant and postpartum mothers and newborns in Finland. The Finnish Population Register Centre (FPRC), established in 1969, includes name, personal identity code, address, citizenship, native language, family members, and date of birth and death (if applicable). It is responsible for usage and maintenance of basic demographic information of all residents in Finland, and was used to identify the controls and to obtain the information on the subjects’ parents and places of birth.

The FiPS-S study received ethical approval from the Ethics Committee of the Hospital District of Southwest Finland, the data protection authorities at the National Institute for Health and Welfare, and the Institutional Review Board of the New York State Psychiatric Institute.

### 2.3. Information on cases and controls

The cases of schizophrenia were born between January 1987 and December 1997 and were registered in the CRHC with ICD-10 (F20) and ICD-9 (295) diagnoses before 2017. The controls were born in Finland, were without a diagnosis of schizophrenia and matched by a ratio of 1:1 to the respective cases on date of birth (±30 days) and sex. The matched controls were alive and residing in Finland at the date of diagnosis of the matched case. Serum specimens with sufficient quantities of sera were available in the FMC for 1145 cases and 1145 matched controls, which were randomly selected from among the whole schizophrenia cohort (1987–1997, *N* = 1672 case-control pairs).

### 2.4. Maternal serum 25(OH)D measurement

Maternal serum 25(OH)D was measured blind to case/control status. Maternal 25(OH)D in archived maternal serum was measured using a chemiluminescence microparticle immunoassay (CMIA) by an Architect i2000SR automatic analyzer (Abbott Diagnostics), which has been described in detail previously (Munger et al., 2016; Sucksdorff et al., 2021). Coefficients of variation were calculated for sample pairs derived from repeated quality control samples included in the assay. The overall assay coefficients of variation percentage calculated from the blinded quality control sample pairs with repeated measures of 25(OH)D included in each set of analyses was 2.1 % indicating the reliability of the assay.

### 2.5. Covariates

The potential confounders and mediators were considered for inclusion in the analyses if they were suggested as having associated with maternal vitamin D levels and offspring schizophrenia in previous literatures (Hauta-Alus et al., 2018; Eggemoen et al., 2016; Miliku et al., 2016; Lokki et al., 2020; Zhou et al., 2017; Niemelä et al., 2016; McGrath et al., 2014; Dean et al., 2010). FMBR was used to obtain the information on maternal and child factors including maternal smoking, maternal SES, maternal age, number of previous births, gestational age, birth weight, and Apgar score at 1 min. FCPR was used to obtain information on the maternal immigrant status, and the season of blood collection and gestational week of blood draw was obtained from FMC. The information on maternal and paternal psychiatric diagnoses, schizophrenia diagnosis and maternal diagnosis of substance use disorder were obtained from the CRHC. The detailed information of potential confounders and mediators is included in Supplementary Table 1.

### 2.6. Statistical analysis

We initially examined maternal 25(OH)D as a continuous variable. Since the variable was skewed, it was log-transformed before analyses. We also examined maternal 25(OH)D into quintiles, and the cut points for the quintiles were based on the distribution of maternal 25(OH)D levels in the control group, with the highest quintile defined as the reference group. The three-class clinical categories of maternal 25(OH)D levels were also examined: deficient (25(OH)D < 30 nmol/L), insufficient (25(OH)D 30–49.9 nmol/L) and sufficient levels (25(OH)D > 50 nmol/L), with the sufficient category as the reference group.

Categorically defined potential confounders from previous literatures were tested with Student’s t and F-tests, and they were tested with linear regression for their association with log-transformed maternal 25 (OH)D among controls. Conditional logistic regression models for the matched sets were used to test for the association between potential confounders with schizophrenia. The covariates were selected into the adjusted models if they were associated with both exposure and outcome at *p* < 0.1.

Point and interval estimates of odds ratios (OR) were obtained by fitting conditional logistic regression models for matched pairs. Unadjusted and adjusted ORs and 95 % confidence intervals were calculated for maternal 25(OH)D levels and offspring schizophrenia. We also investigated whether the effect of maternal 25(OH)D on schizophrenia offspring was modified by sex. For this purpose, sex-by-maternal 25(OH) D interaction effect was added to the statistical models. The interaction effects were deemed to be statistically significant based on a *p*-value <0.10.

Statistical significance was based on *P* < 0.05. All statistical analyses were performed with SAS software (SAS 9.4, SAS Institute, Cary, N.C.).

## 3. Results

**Fig. 1.**
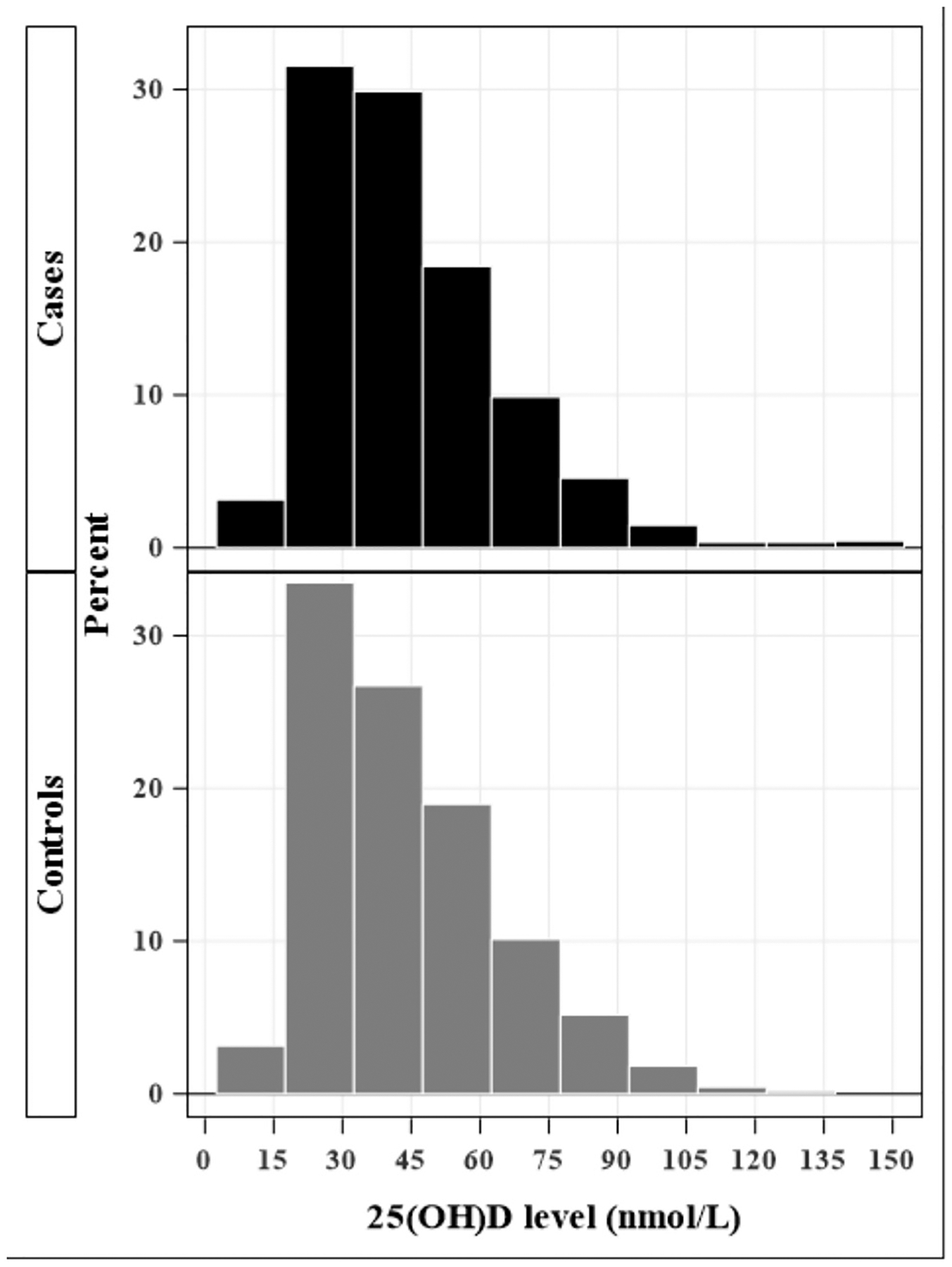
Distribution of maternal 25(OH)D levels among cases and controls.

**Fig. 2.**
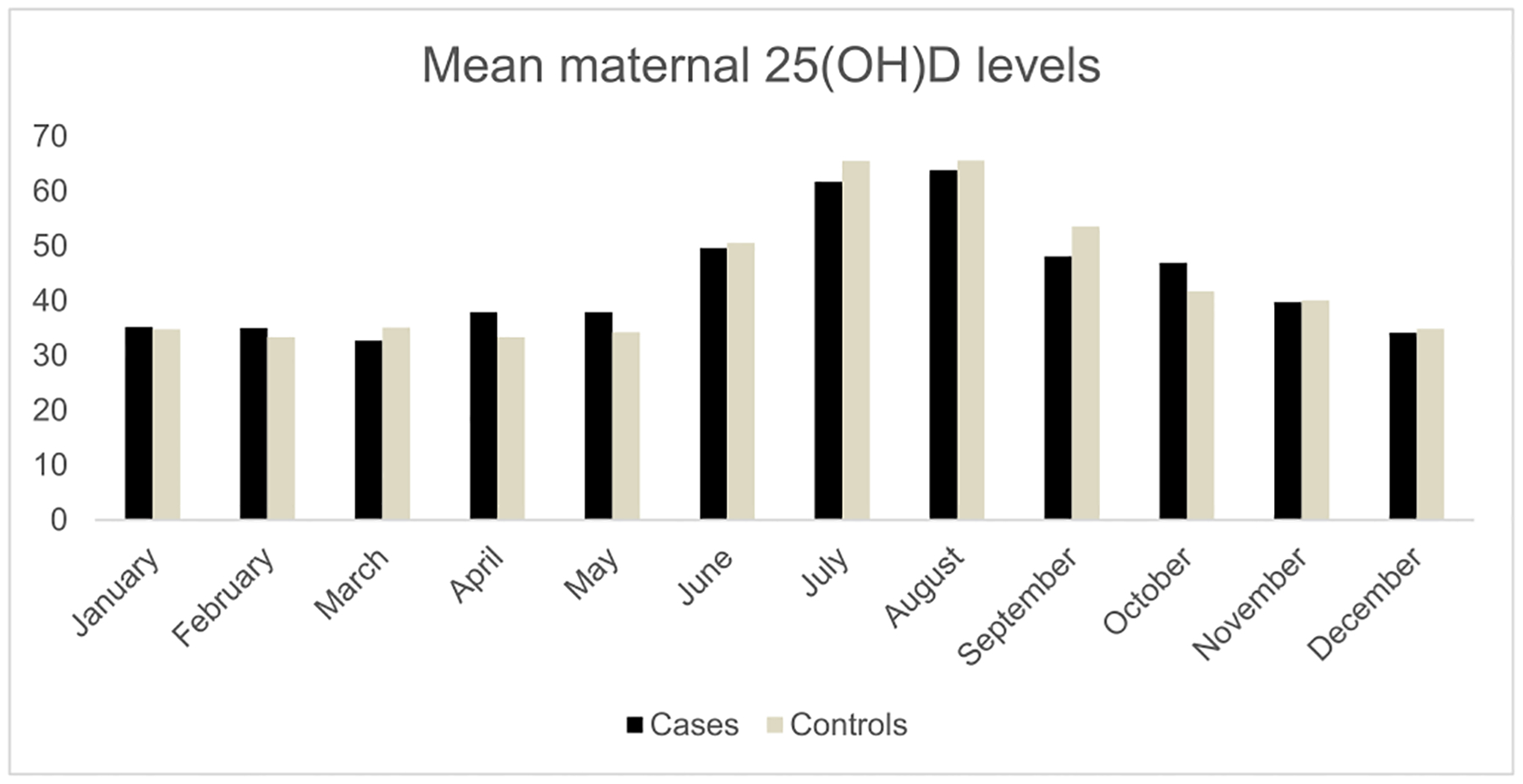
Distribution of monthly maternal 25(OH)D levels among cases and controls.

The study included 1145 case control pairs of schizophrenia with mean age at first schizophrenia diagnosis for cases of 20.63 years (SD: 3.32; range 5–30 years). The median maternal 25(OH)D level among cases was 43.82 nmol/L and 44.06 nmol/L among controls. Fig. 1 shows the distribution of maternal 25(OH)D levels among cases and controls, and Fig. 2 shows the distribution of mean monthly maternal 25(OH)D levels among cases and controls. The mean gestational week of maternal blood draw was 10.87 weeks (range: 4–36 weeks) for cases and 10.15 (range: 4–36 weeks) for controls. The overall sample of cases and controls was 69.34 % male and 30.66 % female.

Supplementary Table 1 shows the association between covariates and maternal 25(OH)D levels among controls, and shows the association between covariates and offspring schizophrenia among case and control subjects. Maternal smoking, history of maternal substance abuse, season of blood collection, season of birth, region of birth, Apgar score, maternal age, paternal age, and gestational week of blood draw were associated with 25(OH)D levels among controls. History of maternal schizophrenia diagnoses, maternal substance abuse, maternal psychopathology, paternal schizophrenia diagnoses, paternal psychopathology, maternal smoking, maternal SES, weight for gestational age, gestational week of blood draw, Apgar score, season of birth, region of birth, paternal immigrant status and maternal immigration status were associated with offspring schizophrenia. Therefore, adjustment was made for maternal smoking, history of maternal substance abuse, season of birth, region of birth, Apgar score and gestational week of blood draw.

Table 1 shows the unadjusted and adjusted results for the association between maternal serum Vitamin D levels and offspring schizophrenia. The maternal 25(OH)D levels, analyzed as a continuous variable, were not associated with offspring schizophrenia in either the unadjusted (OR 0.96, 95 % CI 0.78–1.17, *p* = 0.69) or the adjusted analyses (aOR 0.98, 95 % CI 0.79–1.22, *p* = 0.89). There was no significant association between the lowest quintile of maternal 25(OH)D levels and schizophrenia in either the unadjusted (OR 1.09, 95 % CI 0.81–1.45, *p* = 0.55) or the adjusted analyses (aOR 1.06, 95 % CI 0.78–1.45, *p* = 0.71). Maternal 25(OH)D levels, measured in categories, either in deficient category (OR 1.07 (0.85–1.35), *p* = 0.52; aOR 1.05 (0.81–1.34), *p* = 0.88) or insufficient category (OR 1.13, 95 % CI 0.92–1.40, *p* = 0.23; aOR 1.13, 95 % CI 0.90–1.41, *p* = 0.27) were also not associated with offspring schizophrenia. No evidence of effect modification by sex on the relationship between continuous maternal 25(OH)D and schizophrenia (*p* = 0.35) was found.

## 4. Discussion

Maternal vitamin D levels, measured as circulating 25(OH)D levels in early pregnancy were not associated with the risk of schizophrenia in the offspring in this nationwide population-based study. This is the first study examining maternal vitamin D deficiency in early pregnancy, and thus the hypothesis linking vitamin D deficiency during early pregnancy is not supported by our data.

The finding of our present study is in contrast to previous two Danish register-based studies, that reported positive associations between neonatal vitamin D deficiency and schizophrenia (Eyles et al., 2018; McGrath et al., 2010). It is likely that the proportion of the immigrant population in the Danish studies might have contributed to the associations found in those studies. In both Danish studies, the proportions of the immigrant population were higher compared to the present study, and they reported significantly lower vitamin D levels among offspring of immigrants compared to offspring of native-born parents. Previous studies have suggested an increased risk of schizophrenia in immigrant populations (Henssler et al., 2020) and that individuals with immigrant background have lower vitamin D levels compared to the native population (van der Meer et al., 2011). Furthermore, our study also differs from the Danish studies in the timing of vitamin D deficiency measurement, i.e., we measured vitamin D levels in early pregnancy compared to neonatal sera samples in the previous Danish studies. It is possible that vitamin D deficiency during early pregnancy might not have role in the development of offspring schizophrenia. Interestingly, a study on rats reported altered brain development in the adult rat offspring exposed to vitamin D deficiency in late gestation but not in early gestation (O’Loan et al., 2007). However, it is not clear whether the same applies for humans. Moreover, studies have shown a high correlation between vitamin D levels in early and late pregnancy or at birth (Saraf et al., 2016). Another potential reason for the differences in findings is the fact that only a portion of vitamin D crosses the placenta and thus a more direct measure of vitamin D (neonatal) provides a more accurate index of fetal exposure than maternal levels. Indeed, some studies have shown a relatively weak correlation between maternal and cord blood vitamin D when levels of this nutrient are in the deficient range (Wegienka et al., 2016). Vitamin D supplementation during pregnancy would be expected to improve this correlation. Further studies measuring vitamin D levels during several time points in pregnancy and at birth are needed to draw firm conclusions.

In prior research in this birth cohort, we demonstrated associations between maternal vitamin D levels and two other neurodevelopmental disorders, attention deficit hyperactivity disorder (ADHD) (Sucksdorff et al., 2021) and autism spectrum disorders (ASD) (Sourander et al., 2021). In an animal model, developmental vitamin D deficiency was shown to cause neurotransmitter changes in the glutamatergic, noradrenergic, serotoninergic, and dopaminergic systems, which are implicated in many neuropsychiatric and neurodevelopmental disorders (Kesby et al., 2017). While that model was consistent with some of the neurotransmitter abnormalities, found in schizophrenia, anomalies of neurotransmitter function, including the dopaminergic system, have also been observed in models of ADHD (Madhyastha et al., 2024) and ASD (Lu et al., 2024).

The strengths of the present study include the use of prospectively collected sera in the first and early second trimesters, large sample size for schizophrenia cases, comprehensive information on a range of potential confounders including maternal psychiatric history, immigration background, child’s weight for gestational age, date and place of birth, and prenatal nicotine exposure. There are several limitations that should be considered while interpreting the findings. First, the schizophrenia cases in this study included only offspring who had been referred to specialized services and thus likely represent more severe cases of schizophrenia. Second, maternal 25(OH) D was assessed only at one time point (in the first and early second trimesters) during pregnancy and thus fluctuations in vitamin D levels across pregnancy could not be accounted for. Third, unmeasured factors that may be related to vitamin D levels, and to schizophrenia, such as maternal body mass index, prenatal vitamin supplementation, maternal medications during pregnancy, poor nutrition or lack of compliance with recommended prenatal care, and other maternal behavioral factors were not accounted for, nor was there inclusion of genetic variants; however, we accounted for family history of schizophrenia and other psychopathology, and each of these covariates are unlikely to account for the present findings since they are more likely to be related to reduced maternal vitamin D levels and increased risk of schizophrenia. Study that utilized the Mendelian randomization approach supports the postulate that genetic variants associated with schizophrenia, depression and bipolar disorder are also associated with decreased 25 (OH) D levels (Revez et al., 2020). Although this finding also cannot explain the present results, other observational case-control studies showing positive relationships between maternal vitamin D and schizophrenia may be at least partially due to confounding from that association. We also note that adjustment was made for other several related maternal factors, including smoking, age, substance use, and immigrant status.

## 5. Conclusion

The present study does not support the hypothesis that vitamin D deficiency in early pregnancy is an etiological factor for offspring schizophrenia. Studies with larger sample sizes and longitudinal designs with long follow-up periods are needed to draw firm conclusions. Further studies should measure vitamin D levels at multiple time points during pregnancy, and at birth, to ascertain critical developmental periods where vitamin D deficiency during several stages of pregnancy may be differentially associated with schizophrenia.

## Supplementary Material

Supplementary Material

## Figures and Tables

**Table 1:** Odds ratios and 95 % CI of the association between maternal serum vitamin D and offspring schizophrenia.

Maternal serum vitamin D (nmol/L)	Cases (n = 1145) Mean (SD)	Controls (n = 1145) Mean (SD)	Odds ratio (Unadjusted) 95 % CI	P-value	^a,b^Odds ratio (Adjusted) 95 % CI	P-value
Log transformed maternal vitamin D	43.82 (20.75)	44.06 (20.29)	0.96 (0.78–1.17)	0.69	0.98 (0.79–1.22)	0.89
Quintiles	Cases n (%)	Controls n (%)				
<20	221 (19.30)	226 (19.74)	1.09 (0.81–1.45)	0.55	1.06 (0.78–1.45)	0.71
20–39	214 (18.69)	231 (20.17)	1.03 (0.77–1.36)	0.83	1.00 (0.74–1.35)	0.28
40–59	251 (21.92)	228 (19.91)	1.22 (0.93–1.61)	0.14	1.21 (0.90–1.62)	0.27
60–79	250 (21.83)	230 (20.09)	1.20 (0.92–1.57)	0.16	1.26 (0.95–1.66)	0.12
≥80	209 (18.25)	230 (20.09)	Ref		Ref	
Categories	Cases n (%)	Controls n (%)				
Deficient (<30)	332 (29)	333 (29.08)	1.07 (0.85–1.35)	0.52	1.05 (0.81–1.34)	0.88
Insufficient (30–<50)	456 (39.83)	433 (37.82)	1.13 (0.92–1.40)	0.23	1.13 (0.90–1.41)	0.27
Sufficient (≥50)	357 (31.18)	379 (33.10)	Ref		Ref	

a Adjusted for maternal smoking, history of maternal substance abuse, season of birth, region of birth, Apgar score at 1 min and gestational week of blood draw.

b 1092 case control pairs included in the adjusted model.
