# Supplementary material for: Maternal vitamin D levels during pregnancy and offspring schizophrenia
Source: Schizophr Res. Author manuscript; Available in PMC 2024 Sep 19. (PMC11412259; doi:10.1016/j.schres.2024.06.039)
Supplement: Supplementary Material [file NIHMS2021483-supplement-Supplementary_Material.docx]

**Supplementary Table 1. Relationship between covariates and maternal serum vitamin D among controls, and covariates and schizophrenia in case and control subjects**

|  | **Covariates and maternal serum vitamin D among controls** | | | **Covariates and schizophrenia in case and control subjects** | | | | |
| --- | --- | --- | --- | --- | --- | --- | --- | --- |
|  | **Mean** | **SD** | **P- value** | **Cases**  **n=1145** | | **Controls**  **n=1145** | | **P-value** |
|  |  |  |  | **n** | **%** | **n** | **%** |  |
| **Maternal smoking^1^** |  |  | **<0.001** |  |  |  |  | **0.04** |
| No | 3.71 | 0.44 |  | 903 | 80.63 | 946 | 83.79 |  |
| Yes | 3.51 | 0.43 |  | 217 | 19.38 | 183 | 16.21 |  |
| **Previous births^2^** |  |  | 0.22 |  |  |  |  | 0.14 |
| 0 | 3.70 | 0.45 |  | 435 | 38.22 | 472 | 41.22 |  |
| ≥ 1 | 3.67 | 0.44 |  | 703 | 61.78 | 673 | 58.78 |  |
| **History of maternal**  **psychopathology**^a^ |  |  | 0.19 |  |  |  |  | **<0.001** |
| No | 3.69 | 0.44 |  | 852 | 74.41 | 1000 | 87.34 |  |
| Yes | 3.63 | 0.48 |  | 293 | 25.59 | 145 | 12.66 |  |
| **History of maternal schizophrenia or schizoaffective disorder^d^** |  |  | 0.27 |  |  |  |  | **<0.001** |
| No | 3.68 | 0.45 |  | 1105 | 96.51 | 1140 | 99.56 |  |
| Yes | 3.46 | 0.65 |  | 40 | 3.49 | 5 | 0.44 |  |
| **History of paternal psychopathology^b^** |  |  | 0.65 |  |  |  |  | **<0.001** |
| No | 3.68 | 0.45 |  | 862 | 75.28 | 967 | 84.45 |  |
| Yes | 3.67 | 0.45 |  | 283 | 24.72 | 178 | 15.55 |  |
| **History of paternal schizophrenia or schizoaffective disorder^d^** |  |  | 0.21 |  |  |  |  | **<0.001** |
| No | 3.68 | 0.45 |  | 1115 | 97.38 | 1143 | 99.83 |  |
| Yes | 4.07 | 0.21 |  | 30 | 2.62 | 2 | 0.17 |  |
| **Maternal SES** |  |  | 0.11 |  |  |  |  | **0.002** |
| Upper white collar | 3.75 | 0.44 |  | 98 | 8.56 | 113 | 9.87 |  |
| Lower white collar | 3.70 | 0.42 |  | 305 | 26.64 | 362 | 31.62 |  |
| Blue collar | 3.66 | 0.46 |  | 146 | 12.75 | 123 | 10.74 |  |
| Others | 3.62 | 0.46 |  | 120 | 10.48 | 91 | 7.95 |  |
| Missing | 3.66 | 0.46 |  | 476 | 41.57 | 456 | 39.83 |  |
| **History of maternal substance abuse^c^** |  |  | **0.004** |  |  |  |  | **0.001** |
| No | 3.69 | 0.44 |  | 1069 | 93.36 | 1102 | 96.24 |  |
| Yes | 3.49 | 0.48 |  | 76 | 6.64 | 43 | 3.76 |  |
| **Gestational age (weeks)^3^** |  |  | 0.31 |  |  |  |  | 0.31 |
| <37 | 3.74 | 0.37 |  | 60 | 5.56 | 54 | 4.91 |  |
| ≥37 | 3.68 | 0.45 |  | 1020 | 94.44 | 1046 | 95.09 |  |
| **Weight for gestational age^5^** |  |  | 0.85 |  |  |  |  | **0.06** |
| <-2 SD | 3.62 | 0.51 |  | 52 | 4.59 | 31 | 2.71 |  |
| -2 SD to +2 SD | 3.68 | 0.45 |  | 1038 | 91.53 | 1065 | 93.01 |  |
| >+2 SD | 3.67 | 0.41 |  | 44 | 3.88 | 49 | 4.28 |  |
| **Maternal immigration status** |  |  | 0.20 |  |  |  |  | **0.01** |
| No | 3.68 | 0.45 |  | 1127 | 98.43 | 1140 | 99.56 |  |
| Yes | 3.93 | 0.61 |  | 18 | 1.57 | 5 | 0.44 |  |
| **Paternal immigration status^6^** |  |  | 0.75 |  |  |  |  | **0.03** |
| No | 3.68 | 0.45 |  | 1104 | 97.96 | 1125 | 99.12 |  |
| Yes | 3.73 | 0.49 |  | 23 | 2.04 | 10 | 0.88 |  |
| **Season of blood collection** |  |  | **<0.001** |  |  |  |  | 0.31 |
| Winter | 3.46 | 0.38 |  | 292 | 25.50 | 293 | 25.59 |  |
| Spring | 3.47 | 0.40 |  | 263 | 22.97 | 266 | 23.23 |  |
| Summer | 4.03 | 0.36 |  | 289 | 25.24 | 309 | 26.99 |  |
| Autumn | 3.72 | 0.39 |  | 301 | 26.29 | 277 | 24.19 |  |
| **Season of birth** |  |  | **<0.001** |  |  |  |  | **0.04** |
| April-November | 3.55 | 0.41 |  | 744 | 64.98 | 764 | 66.72 |  |
| December-March | 3.93 | 0.41 |  | 401 | 35.02 | 381 | 33.28 |  |
| **Region of birth^7^** |  |  | **0.03** |  |  |  |  | **<0.001** |
| Urban | 3.70 | 0.44 |  | 772 | 67.60 | 670 | 58.62 |  |
| Semi-urban | 3.66 | 0.46 |  | 165 | 14.45 | 217 | 18.99 |  |
| Rural | 3.61 | 0.46 |  | 205 | 17.95 | 256 | 22.40 |  |
| **Apgar Score at 1 min^4^** |  |  | **0.004** |  |  |  |  | **0.05** |
| 0-6 | 3.61 | 0.51 |  | 43 | 3.80 | 33 | 2.88 |  |
| 7-8 | 3.65 | 0.45 |  | 235 | 20.76 | 202 | 17.64 |  |
| 9-10 | 3.69 | 0.45 |  | 854 | 75.44 | 910 | 79.48 |  |
|  | **Beta** | **SE** |  | **Mean** | **SD** | **Mean** | **SD** |  |
| **Maternal age (years)** | 3.42 | 0.07 | **<0.001** | 28.82 | 5.58 | 28.78 | 5.07 | 0.84 |
| **Paternal age (years)^8^** | 3.45 | 0.07 | **<0.001** | 31.36 | 6.08 | 30.93 | 5.84 | 0.11 |
| **Gestational week of blood draw^9^** | 3.83 | 0.04 | **<0.001** | 10.87 | 4.0 | 10.15 | 3.29 | **<0.001** |

^a^ ICD-8 (291-308), ICD-9 (291-316) or ICD-10 (F10-99), excluding intellectual disability (F70-79) and excluding maternal substance abuse diagnosis, ^b^ ICD-8 (291-308), ICD-9 (291-316) or ICD-10 (F10-99), excluding intellectual disability (F70-79); ^c^ ICD-8 (291, 303, 304), ICD-9 (291, 292, 303,304,305) or ICD-10 (F10-19); ^d^ (ICD-10 F20-25 or 295 in ICD 9) SD: standard deviation; SE Sandard Error; SES Socio-economic status

^1^ Data missing for 25 cases and 16 controls; ^2^ Data missing for 7 cases; ^3^ Data missing for 65 cases and 45 controls; ^4^ Data missing for 13 cases; ^5^ Data missing for 11 cases; ^6^ Data missing for 28 cases; ^7^ Data missing for 3 cases and 2 controls; ^8^Data missing for 18 cases and 10 controls; ^9^ Data missing for 11 cases.
